# Small hole in the skin with an unexpected image in ultrasound

**DOI:** 10.1016/j.jdcr.2023.09.010

**Published:** 2023-09-24

**Authors:** Ariany T.A.S. Denofre, Ana Carolina B. Silva, Carolina M. Stecca, Renata F. Magalhães, Thais H. Buffo

**Affiliations:** Dermatology Division, University of Campinas – Unicamp, São Paulo, Brazil

**Keywords:** dermatological ultrasound, infectious disease

A 57-year-old woman with Xeroderma pigmentosum comes for routine follow-up and describes the appearance of a 2 cm whitish and painful nodule on her left leg with a small hole at the top (Fig 1). She has experienced itchiness and a stinging sensation for 2 weeks. The patient did not report any mosquito bites. Dermoscopy evaluation revealed a small hole within the initial hole (Fig 2). The patient had previously received treatment for 7 melanomas and 15 basal cell carcinomas. Considering the high risk of cutaneous metastasis, we requested a dermatologic ultrasound (Fig 3) to aid in the diagnosis. A linear hyperchoic structure with a positive Doppler sign was observed. Occlusion with Vaseline and mechanical expression resulted in the removal of a larva (Fig 4).

**Fig 1:**
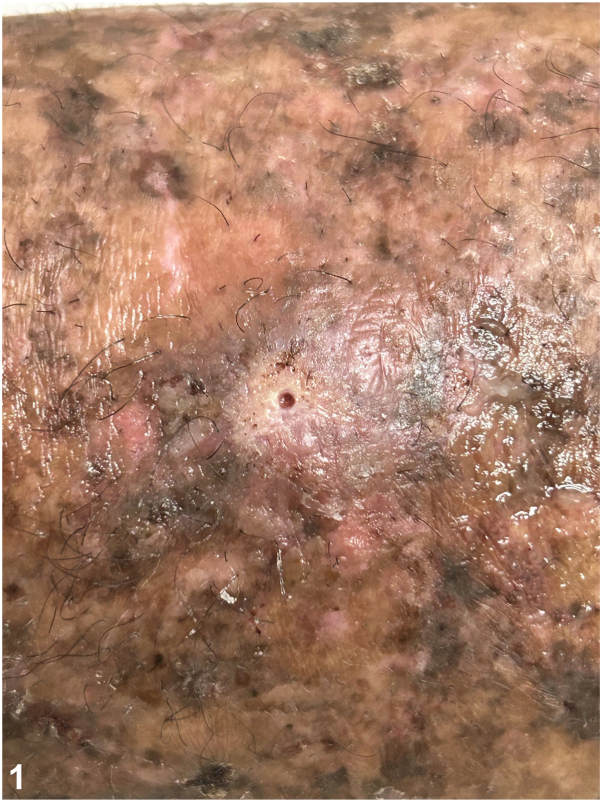

**Fig 2:**
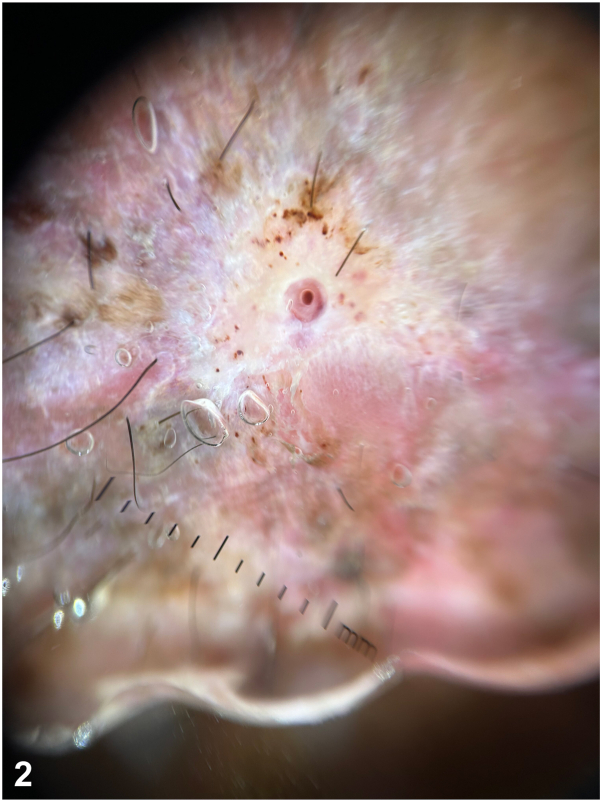

**Fig 3:**
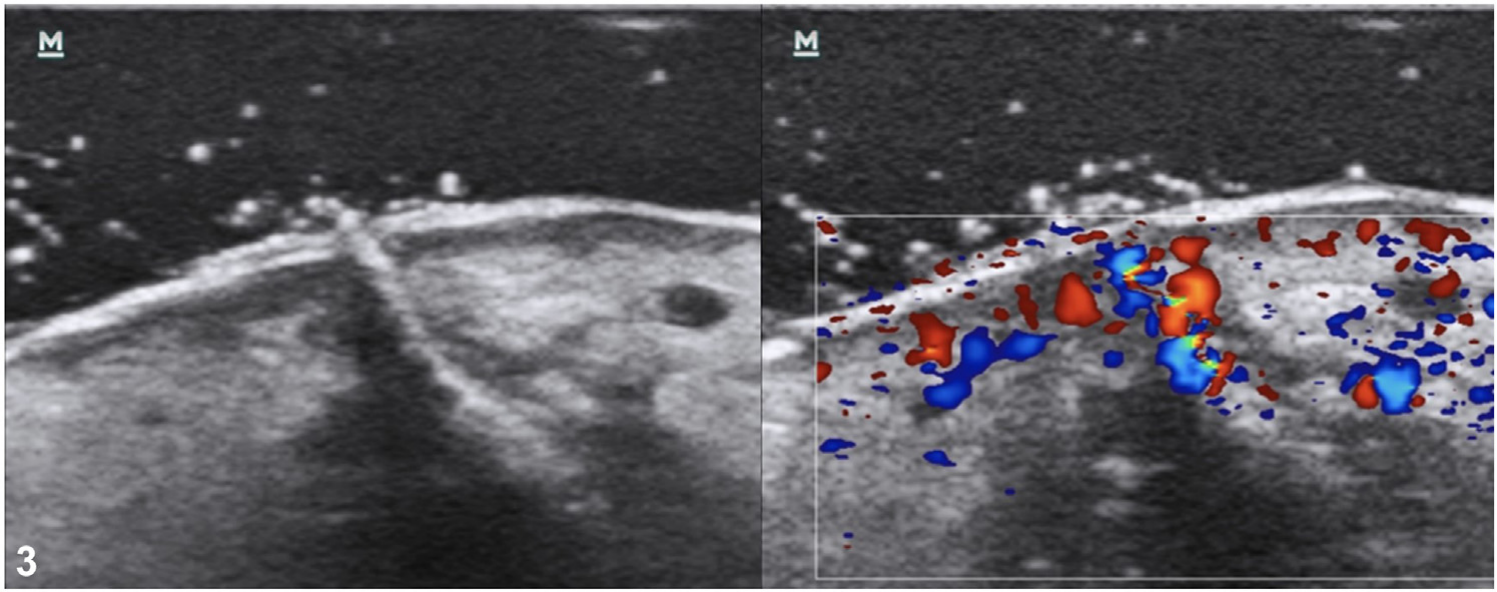

**Fig 4:**
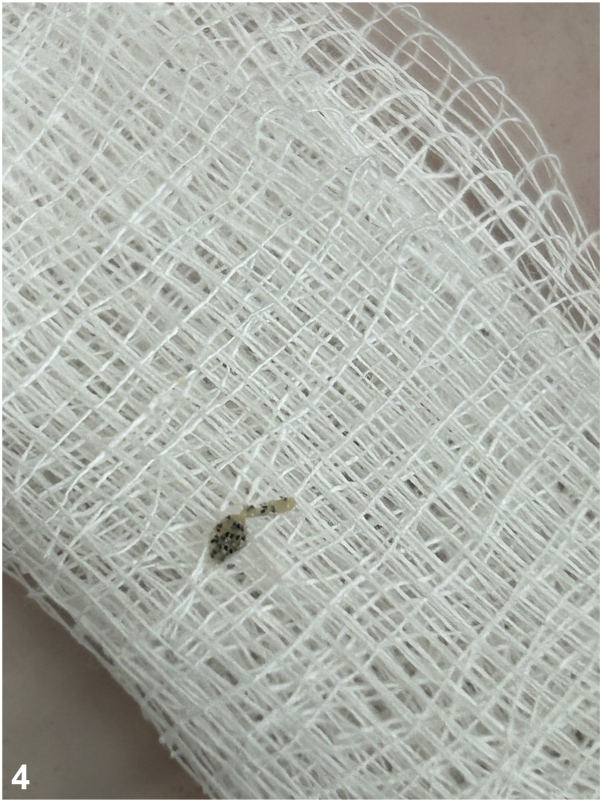

**Question 1: Considering the clinical presentation, dermoscopy, and ultrasonographic findings, what is the diagnosis?**

A. Epidermoid cyst
B. Myiasis
C. Furunculosis
D. Tungiasis
E. Bedbug

**Answers:**

A. Epidermoid cyst – Incorrect. The epidermoid cysts originate from the follicular infundibulum and contain keratinous fluid. Although it also has a small hole on the top, the punctum, it normally presents with black keratin. The ultrasound image corresponds to an oval-shaped ultrasound structure with a central hypoechoic band.
B. Myiasis – Correct. Myiasis corresponds to the invasion of the tissue by dipterous larvae. It occurs worldwide, with a higher prevalence in tropical and subtropical regions. In nontropical areas, it corresponds to the fourth most common travel-associated skin disease. There are 2 types of myiasis: furunculous and cavitary.^1^^,^^2^
C. Furunculosis – Incorrect. Normally caused by Staphylococcus aureus, furunculosis presents as a papule or nodule with erythema and pus.
D. Tungiasis – Incorrect. Tungiasis appears as a yellow papule with a brown center, normally on the feet. The dermoscopy shows the structures of the flea.^3^
E. Bedbug – Incorrect. Bedbugs are blood-sucking parasites that live on mattresses and sheets worldwide. They are active at night when they bite the skin, resulting in multiple erythematous papules that are itchy.

**Question 2: Which agent is responsible for this type of disease?**

A. *Lutzomia longipalpis*
B. *Cochliomyia hominivorax*
C. *Ancylostoma brasiliensis*
D. *Dermatobia hominis*
E. *Madurella mycetomatis*

**Answers:**

A. *Lutzomia longipalpis* – Incorrect. This agent is a mosquito that is responsible for the transmission of *Leishmania sp.*, causing the ulcer of Leishmaniasis.
B. *Cochliomyia hominivorax* – Incorrect. *Cochliomyia hominivorax* is a fly whose larvae cause cavitary myiasis. It affects tumors, wounds, and necrotic areas with poor hygiene.
C. *Ancylostoma brasiliensis* – Incorrect. Ancylostoma brasiliensis causes cutaneous larva migrans, a filariform larva disease caused by contact with contaminated soil by dog and cat feces. It is the most common skin disease reported by travelers returning from tropical regions.
D. *Dermatobia hominis* – Correct. *Dermatobia hominis* is distributed mainly in Central and South America, and it is more common in rural areas. This butterfly has red eyes, a yellow face, and a metallic blue and orange midsection. Infestation occurs in humans, cattle, and other mammals. The female bumblebee puts its eggs in a mosquito, fly, or tick, and this agent transmits the egg to the mammal, which grows in the skin.^4^^,^^5^
E. *Madurella mycetomatis* – Incorrect. Madurella mycetomatis and other agents like *Nigrograna mackinnonii and Trematosphaeria grisea* cause Eumicetoma, a chronic fungus infection that forms tumors and fistulae and eliminates small granules in the lower limbs.

**Question 3: What is the necessary treatment for these cases?**

A. Occlusion + extraction
B. Ivermectin
C. Albendazole
D. Surgery
E. Extraction

**Answers:**

A. Occlusion + extraction – Correct. Apply petrolatum, nail polish, bee wax, or anything that should kill the maggot by hypoxia for 3-24 hours, and then mechanical expression is the main successful treatment.
B. Ivermectin – Incorrect. Ivermectin is not necessary, as the maggots have hypoxia within a few hours, making it easily extracted. Besides that, it could be used orally before the mechanical extraction.
C. Albendazole – Incorrect. Albendazole is an anthelminthic agent used in cutaneous larva migrans and other similar diseases that has no effect on myiasis.
D. Surgery – Incorrect. Surgery is too aggressive for infections that can simply be resolved with occlusion and mechanical expression.
E. Extraction – Incorrect. As the larvae have a hocked mouth, it is almost impossible to extract them without hypoxia. Forced mechanical extraction facilitates infection and the remaining parts of the larvae inside the body.

## Conflicts of interest

None disclosed.

## References

[1] Davis C.A., Patterson J., Hampton K.A. (2022). Point of care ultrasound findings in a case of botfly myiasis contracted in the US. Wilderness Environ Med.

[2] Papineni V., Dieu S., Rennie W.J. (2023). The human botfly “bubbling sign”: ultrasound features of cutaneous furuncular myiasis. Indian J Radial Imaging.

[3] Bakos R.M., Reinehr C., Escobar G.F., Leite L.L. (2021). Dermoscopy of skin infestations and infections (entomodermoscopy) – part I: dermatozoonoses and bacterial infections. An Bras Dermatol.

[4] Ragi S.D., Kapila R., Schwartz R.A. (2021). The botfly, a tropical menace: a distinctive myiasis caused by dermatobia hominis. Am J Clin Dermatol.

[5] Renato M., Bakos L. (2007). Dermatoscopic diagnosis of furuncular myiasis. Arch Dermatol.

